# A nomogram for predicting unfavorable outcomes of antituberculosis treatment among individuals with AIDS combined with pulmonary tuberculosis in China

**DOI:** 10.3389/fimmu.2025.1594107

**Published:** 2025-05-29

**Authors:** Xiaoxu Han, Jin Sun, Yuan Gao, Hongxia Yan, Xiangchuan He, Yuanyuan Ma, Peng Xu, Ning Ding, Xin Zhang, Meixin Ren, Taiyi Jiang, Tong Zhang, Bin Su

**Affiliations:** ^1^ Beijing Key Laboratory for HIV/AIDS Research, Clinical and Research Center for Infectious Diseases, Beijing Youan Hospital, Capital Medical University, Beijing, China; ^2^ Sino-French Joint Laboratory for HIV/AIDS Research, Sino-French Joint Laboratory for Research on Humoral Immune Response to HIV Infection, Beijing Youan Hospital, Capital Medical University, Beijing, China; ^3^ Scientific and Technological Achievement Transformation Center, Beijing Youan Hospital, Capital Medical University, Beijing, China; ^4^ Central Laboratory, Beijing Youan Hospital, Capital Medical University, Beijing, China

**Keywords:** AIDS, pulmonary tuberculosis, antituberculosis treatment, treatment outcome, LASSO Cox model, nomogram, risk stratification

## Abstract

**Background:**

Acquired Immune Deficiency Syndrome (AIDS) combined with tuberculosis (TB) is one of the key factors affecting global TB control, and timely and effective treatment is essential to improve the prognosis in this population. However, data from the WHO have shown that patients with AIDS combined with TB have a lower anti-TB treatment success rate than HIV-negative individuals do, which may lead to an increased incidence of treatment relapse and drug resistance. Therefore, exploring the risk factors affecting the outcome of anti-TB treatment in patients with AIDS combined with TB and developing relevant predictive models will help clinicians rapidly identify patients at greater risk of treatment failure, which is highly valuable for clinical management.

**Methods:**

We conducted a retrospective cohort study including inpatients with AIDS combined with pulmonary tuberculosis (PTB) who were treated at Beijing Youan Hospital between January 2020 and January 2024. The baseline data and laboratory test data of all enrolled patients were collected from the electronic medical records system. We randomly divided the participants into a training set and a validation set at a ratio of 2:1 and established a LASSO Cox model on the basis of the training set to identify risk factors affecting the outcome of anti-TB treatment. The selected prognostic factors were then used to construct the final Cox model, which was visualized using a nomogram. The receiver operating characteristic (ROC) curves, concordance index (C-index), and calibration curves of the training set and validation set were used to evaluate the discrimination ability and consistency of the model, respectively. Decision curve analysis (DCA) was used to assess the clinical applicability of the prognostic models. Patients were subsequently risk stratified according to the optimal cutoff value selected by X-tile software for better clinical decision-making by clinicians.

**Results:**

A total of 203 inpatients with AIDS combined with PTB were enrolled in this study, including 141 (69.5%) with treatment success and 62 (30.5%) with unfavorable outcome. The results of the LASSO Cox regression model revealed that the CRP/albumin ratio (CAR), extrapulmonary disseminated tuberculosis, other pulmonary infectious diseases, and pulmonary cavitation were independent risk factors for unfavorable outcomes in patients with AIDS combined with PTB, whereas the CD4^+^ T-cell counts was a protective factor affecting patient outcomes. The five variables in the final Cox regression model were further used to establish a predictive nomogram. The AUC (0.760 for the training set and 0.811 for the validation set) and C-index (0.765 for the training set and 0.768 for the validation set) showed that the model we constructed had good discrimination ability. The calibration curves indicated high consistency between the predictions and the actual observations in both the training set and the validation set. DCA for the training set and validation set revealed that the nomogram had clinical applicability. Patients were risk-stratified according to the total nomogram score, and the patients were divided into three groups: low risk (total points <358), medium risk (358 ≤ total points <373), and high risk (total points ≥373). Clinicians should focus on patients whose total score is more than 358 points.

**Conclusion:**

We identified prognostic factors for unfavorable anti-TB treatment outcomes and constructed a predictive nomogram to assess the risk of treatment failure in patients with AIDS combined with PTB. Our model performed satisfactorily and can be used for the clinical screening and management of high-risk patients.

## Introduction

Tuberculosis (TB) is the leading cause of death from a single infectious agent, which places a heavy burden on health care systems worldwide. The combination of Acquired Immune Deficiency Syndrome (AIDS) and TB is a priority for TB outbreak prevention and control. Compared with people who are not infected with human immunodeficiency virus (HIV), people who are infected with HIV are approximately 14 times more likely to develop TB (1). *Mycobacterium tuberculosis (Mtb)* infection can accelerate the progression of AIDS by facilitating HIV replication and cell-to-cell transmission of the virus. Moreover, HIV infection can increase the likelihood of endogenous relapse and exogenous reinfection with *Mtb* (2). These two pathogens mutually promote disease progression, rapidly leading to death (3). According to the *Global Tuberculosis Report 2024* (4), there were 10.8 million new cases of TB worldwide in 2023, with 662,000 TB patients coinfected with HIV. TB resulted in the death of 1.25 million people, including 161,000 HIV-infected individuals, accounting for 12.9% of all TB-related deaths. Therefore, timely and effective anti-TB treatment is crucial.

Owing to their compromised immune status and the additive side effects of concomitant medications, HIV-infected individuals are more likely to experience anti-TB treatment failure (5). A history of anti-TB treatment significantly increases the risk of developing drug-resistant tuberculosis (DR-TB), ultimately leading to increased mortality rates (6). In 2023, the global treatment success rate for HIV/*Mtb*-coinfected patients was 79%, which was lower than the 88% success rate for patients without HIV infection (4). Studies indicate that the mortality rate of patients with AIDS combined with TB during treatment is twice as high as that of people without HIV infection (1). Even more alarmingly, the mortality risk among AIDS patients with DR-TB is four times greater than that among those without HIV infection (7). Therefore, identifying the key factors contributing to treatment failure is essential for increasing the success rate of tuberculosis treatment and advancing the goals of the WHO-End TB Strategy (8).

The least absolute shrinkage and selection operator (LASSO) Cox model performs both feature selection and survival prediction, identifying key factors that influence patient survival. Compared to other models such as the Random Forest model and the Neural Network model, the LASSO Cox model can be directly transformed into a nomogram for visualizing individualized risk scores, which is more convenient for clinical application (9). The nomogram is a graphical prediction tool that integrates multiple parameters to estimate patient survival outcomes and is widely used in medical prognostic research. Previous study showed that nomogram models are widely used to establish clinical prognostic models for TB and multidrug-resistant tuberculosis (MDR-TB) (10–14). Risk factors associated with unfavorable anti-TB treatment outcomes include age, sex, smoking status, alcohol abuse status, diabetes status, undernutrition status, and treatment history (15–19). However, most previous studies have focused on TB patients, and the risk factors associated with anti-TB treatment failure in patients with AIDS combined with pulmonary tuberculosis (PTB) remain unclear. Therefore, we collected comprehensive data on individuals with AIDS combined with PTB, including laboratory indicators and clinical visit records. Our goal was to identify risk factors contributing to unfavorable outcomes using the LASSO Cox model and construct a predictive nomogram that will help clinicians quickly identify patients at greater risk of treatment failure.

## Methods

### Subjects

**Figure 1:**
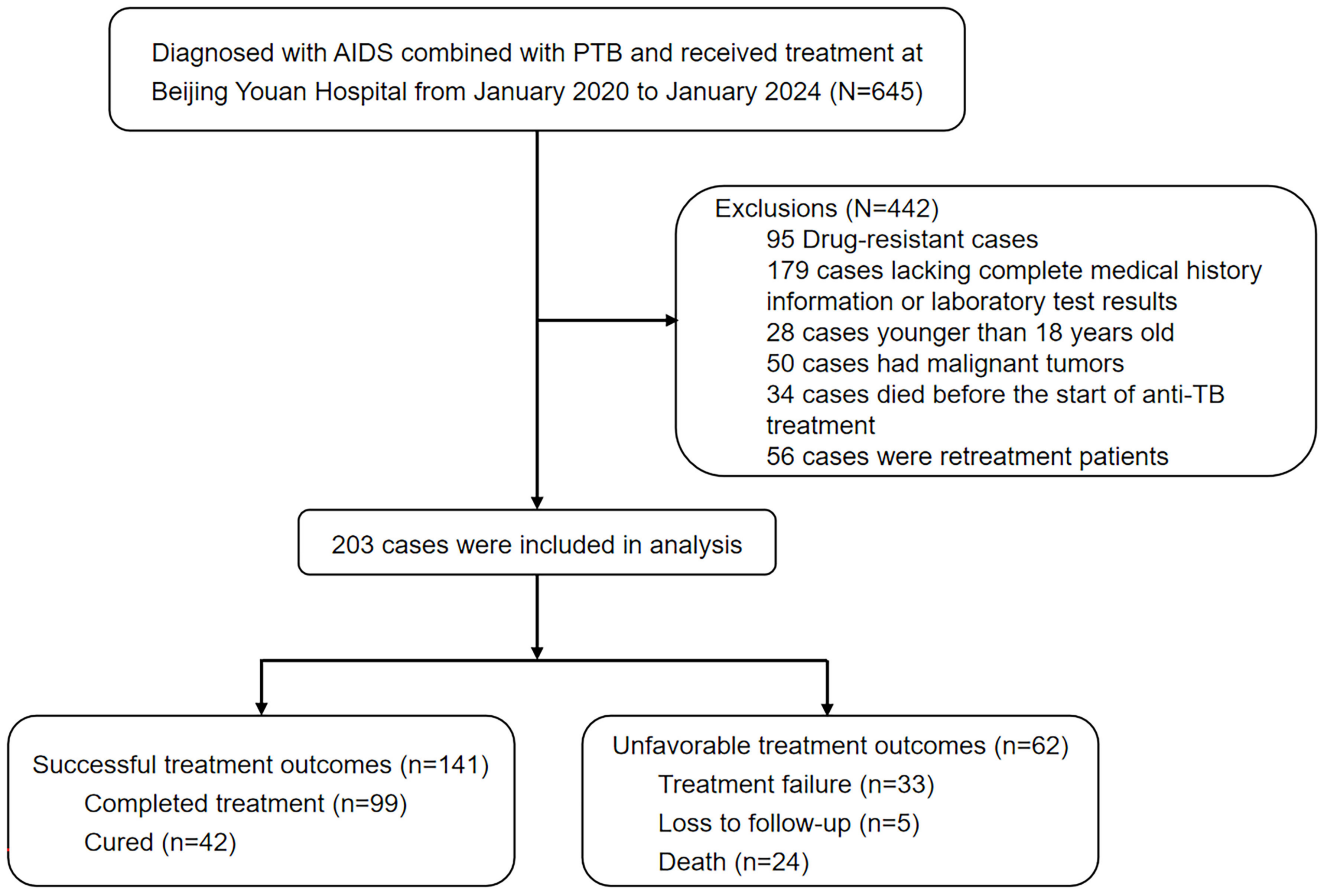
Flowchart of the inclusion and exclusion criteria for the study participants.

We conducted a retrospective study in an observational cohort. We enrolled inpatients who were diagnosed with AIDS combined with PTB and who received treatment in the Clinical and Research Center for Infectious Diseases at Beijing Youan Hospital from January 2020 to January 2024. All enrolled participants received a rifampicin/rifabutin-based standard anti-TB regimen (combination of isoniazid, rifampicin/rifabutin, ethambutol, and pyrazinamide for 2 months intensive treatment, followed by a continuation phase with isoniazid plus rifampicin/rifabutin for 4 months). The duration of treatment may be extended to 9–12 months for patients combined with central nervous system tuberculosis and 6–9 months for patients with bone or joint tuberculosis. The course of anti-TB treatment for patients with other extrapulmonary tuberculosis was usually 6 months. At the beginning of treatment, all patients were hospitalized to receive anti-TB treatment. At the time of discharge, patients were educated on dosage and frequency of medication, medication adherence, and possible drug side effects and management methods. Patients are given instructions to follow up regularly to monitor the development of drug resistance and adverse effects. Our study focused on this population because standard anti-TB treatment regimens are able to respond to the most common TB management conditions in China. The specific inclusion criteria were as follows: (1) the diagnosis and management of HIV/AIDS were in line with the *Chinese guidelines for the diagnosis and treatment of human immunodeficiency virus infection/acquired immunodeficiency syndrome (2024 edition)* (20); (2) the diagnosis of PTB was confirmed by etiological testing, molecular testing or next-generation sequencing (NGS) testing; and (3) no prior anti-TB treatment or treatment for less than 1 month. The exclusion criteria were as follows: (1) were younger than 18 years old, (2) had rifampicin and/or isoniazid-resistant TB, (3) died before the start of anti-TB treatment, (4) were coinfected with nontuberculous mycobacteria infection, (5) had malignant tumors, and (6) had incomplete medical history information. **Figure 1** showed a flowchart of the criteria for inclusion and exclusion of participants.

### Ethics statement

This study was approved by the Beijing Youan Hospital Research Ethics Committee (No. 2018-025, 2021-275, 2023-020, 2024-097). This study was in accordance with the Declaration of Helsinki. Because of the retrospective nature of the study, the Research Ethics Committee waived the requirement for informed consent. The methods in the study conformed to approved guidelines and regulations.

### Data collection

We collected the baseline data of all individuals from an electronic medical record system, including basic demographic information (such as sex and age), the status of extrapulmonary disseminated infections (including tuberculous pleurisy, central nervous system tuberculosis, bone and joint tuberculosis, and other systems), and complications (hepatitis, syphilis, chronic obstructive pulmonary disease, diabetes, cardiovascular disease, and other pulmonary infectious diseases such as Pneumocystis carinii pneumonia (PCP) and Klebsiella pneumonia). We also recorded each patient’s chest imaging findings. The laboratory data included blood tests (such as tests to determine the hemoglobin (HGB) level, red blood cell (RBC) count, white blood cell (WBC) count, and neutrophil count), liver function tests (alanine aminotransferase (ALT), aspartate transaminase (AST), etc.), inflammatory markers (including the C-reactive protein (CRP) level, erythrocyte sedimentation rate (ESR) and procalcitonin level), and immunological examination results (the CD4^+^ T-cell counts and CD8^+^ T-cell counts). In addition, we recorded a number of combined inflammatory marker indicators, including the neutrophil-to-lymphocyte ratio (NLR), lymphocyte-to-monocyte ratio (LMR), and CRP/albumin ratio (CAR).

### Definitions

According to the *Technical Guidelines for Tuberculosis Control in China* (21) and an updated version of TB treatment outcome determination guidelines published by the WHO (1), the outcomes of anti-TB treatment were categorized as follows: cure, completion of treatment, treatment failure, death during treatment, and loss to follow-up. A cure and treatment completion were considered successful treatment outcomes. A cure was defined as a positive pathogenicity test result in a patient who completed the prescribed course of treatment with bacteriological evidence of sputum bacterial conversion. Treatment completion consisted of two scenarios: (1) a negative pathogenicity test result in a patient who completed the prescribed course of treatment and had negative sputum smears or cultures at the end of the course or had no smear tests; and (2) a positive pathogenicity test result in a patient who completed the prescribed course of treatment and had no tests at the end of the course of treatment but had negative results on the most recent sputum smears or cultures. Treatment failure, death during treatment, and loss to follow-up were considered unfavorable outcomes. Treatment failure meant that patients needed to discontinue treatment or permanently change to a new treatment regimen owing to the lack of a clinical or pathogenetic response, adverse drug reactions, or the acquisition of evidence of drug resistance. Death was defined as death for any reason during anti-TB treatment. Loss to follow-up consisted of the abandonment of treatment, the interruption of treatment for more than 2 months because of patient self-discontinuation or other reasons, or the transfer of a patient to another hospital where their outcome could not be tracked. The outcome event of Cox proportional hazard analysis was defined as unfavorable outcomes occurring within 12 months of anti-TB treatment initiation.

### Statistical analysis

We used Jamovi software (version 2.6.17) for data analysis. Comparisons of baseline characteristics, comorbidities, and laboratory test data were performed between two groups of participants with different outcomes: patients with AIDS combined with PTB who had successful treatment outcomes and patients who had unfavorable outcomes. For continuous variables, normally distributed data are presented as the means ± standard errors, and *t*-tests were used for comparisons between groups. Non-normally distributed data are expressed as median (interquartile range (IQR)), and group comparisons were performed by the Mann-Whitney U test. In addition, categorical variables are presented as frequencies and percentages, and comparisons between groups were performed using the X^2^ test.

R version 4.3.3 was used to construct and validate the nomograms for predicting unfavorable outcomes. First, we constructed a LASSO regression model to determine potential risk factors associated with the occurrence of unfavorable outcomes. The 10-fold cross-validation method was used to choose the optimal parameter λ. Subsequently, nonzero coefficients screened by LASSO regression were incorporated into the Cox model to identify independent predictive factors of unfavorable outcomes. The selected prognostic factors were used to model the final Cox model and visualize the model using a nomogram. Internal validation of the model was performed by repeated sampling 1000 times using the bootstrap repeated sampling method. Receiver operating characteristic (ROC) curves, concordance index (C-index), and calibration curves were used to assess the discrimination ability and consistency of the model, respectively. Decision curve analysis (DCA) was used to evaluate whether our prognostic model could help us make better clinical decisions by quantifying the net benefit within a reasonable risk threshold (22). X-tile software was used to select the optimal cutoff value for the total score of the nomogram (23). A Kaplan–Meier (K–M) curve was used to assess the difference in the probability of unsuccessful treatment outcomes for patients in different risk groups. All the statistical tests were two-sided, and *p* < 0.05 was considered statistically significant.

## Results

### Baseline characteristics of patients with AIDS combined with PTB who had unfavorable treatment outcomes

A total of 203 inpatients with AIDS combined with PTB were included in our retrospective cohort, of whom 195 (96.06%) were males and 8 (3.94%) were females, and the median age of these patients was 38 years (IQR 30–49 years). Among the 203 enrolled participants, 141 (69.5%) had successful treatment outcomes, including 42 (20.7%) who were cured and 99 (48.8%) who completed treatment, whereas 62 (30.5%) patients experienced unfavorable outcomes, including 33 (16.3%) with treatment failure, 24 (11.8%) who died and 5 (2.4%) who were lost to follow-up.

The individuals were divided into two groups on the basis of treatment outcomes, and **Table 1** shows the differences in baseline characteristics between the two groups. The median age of patients with successful treatment outcomes was 38 years (IQR 31–48 years), and 135 (95.7%) patients were males. Among the 141 patients with successful treatment outcomes, 52 (36.9%) had coinfection with extrapulmonary disseminated tuberculosis, 57 (40.4%) had other pulmonary infectious diseases, and 14 (9.9%) had cardiovascular disease. Sixty-two patients met the criteria for unfavorable treatment outcomes, including 60 (96.8%) males, and the median age of these patients was 38 years (IQR 30–51 years). Patients in the unfavorable outcome group had a higher prevalence of extrapulmonary disseminated tuberculosis (64.5%), other pulmonary infections (71.0%), and cardiovascular disease (21.0%) compared to the treatment success group, with *p* values of <0.001, <0.001 and 0.033, respectively. There was no significant difference in the incidence of hepatitis, syphilis, chronic obstructive pulmonary disease, or diabetes. A comparison of the chest imaging features between the two groups of patients revealed that the incidence of miliary nodules and pulmonary cavitation was significantly greater in the treatment failure group than in the treatment success group (*p*=0.042 and *p*=0.036, respectively). Compared with patients with successful treatment outcomes, patients with unfavorable outcomes had lower HGB levels, RBC counts, and albumin levels (*p* values of 0.028, 0.007, and 0.001, respectively), whereas CRP and procalcitonin levels were significantly greater (*p*<0.001 and *p*=0.008, respectively). The NLR, LMR, and CAR are important markers reflecting the inflammatory state of the host and play key roles in the therapeutic assessment and prognosis of important diseases, such as COVID-19, pneumonia, and tumors (24–27). Assessment of the differences in the NLR and CAR between the two groups revealed that patients in the treatment failure group had considerably greater NLR and CAR than did those in the treatment success group (*p*=0.002 and *p*<0.001, respectively). Additionally, patients with unfavorable outcomes had significantly lower CD4^+^ T-cell counts and CD8^+^ T-cell counts than did those with treatment success (*p*<0.001 and *p*<0.001, respectively).

**Table 1:** Baseline characteristics of patients with unfavorable outcomes.

Characteristics	Patients with successful treatment outcomes (n=141)	Patients with unfavorable treatment outcomes (n=62)	Statistic	*p* value
Gender, n (%)				
Male	135 (95.7)	60 (96.8)	0.121	0.728
Female	6 (4.3)	2 (3.2)		
Age (years)	38 (31, 48)	38 (30, 51)	0.036	0.971
Extrapulmonary disseminated infection, n (%)	52 (36.9)	40 (64.5)	13.273	<0.001*
Complications, n (%)				
Hepatitis	9 (6.4)	5 (8.1)	0.190	0.663
Syphilis	29 (20.7)	16 (25.8)	0.644	0.422
Other pulmonary infectious disease	57 (40.4)	44 (71.0)	16.069	<0.001*
Chronic obstructive pulmonary disease	7 (5.0)	4 (6.5)	0.186	0.666
Diabetes	6 (4.3)	5 (8.1)	1.219	0.270
Cardiovascular disease	14 (9.9)	13 (21)	4.551	0.033*
Chest imaging abnormal, n (%)				
Miliary nodules	18 (12.8)	15 (24.2)	4.131	0.042*
Pulmonary cavitation	11 (7.8)	11 (17.7)	4.404	0.036*
Initiate ART, n (%)	73 (51.8)	35 (56.5)	0.379	0.538
ART regimens, n (%)				
2NRTIs+NNRTIs	47 (64.4)	21 (60.0)	0.519	0.972
Elvitegravir	9 (12.3)	5 (14.3)		
2NRTIs+INSTIs	5 (6.8)	3 (8.6)		
2NRTIs+PIs	4 (5.5)	2 (5.7)		
Others	8 (11.0)	4 (11.4)		
Blood biochemical tests				
RBC (1012/L) counts, n (%)				
< 4.0	92 (65.2)	50 (80.6)	4.857	0.028*
≥ 4.0	49 (34.8)	12 (19.4)		
HGB (g/L)				
< 110	76 (53.9)	46 (74.2)	7.395	0.007*
≥110	65 (46.1)	16 (25.8)		
WBC counts (109/L)	5.55 (3.58, 6.75)	4.93 (3.30, 7.72)	-0.136	0.892
Neutrophils (109/L)	3.64 (2.24, 5.07)	3.84 (2.33, 5.84)	1.026	0.305
Lymphocyte (109/L)	0.83 (0.50, 1.24)	0.28 (0.14, 0.62)	-2.889	0.004*
Monocyte (109/L)	0.37 (0.28, 0.51)	0.31 (0.21, 0.46)	-1.860	0.063
Platelet (109/L)	232.00 (181.00, 279.50)	226.34 ± 15.37	-0.824	0.410
K^+^ concentration	4.00 (3.64, 4.31)	4.00 (3.60, 4.35)	-0.057	0.954
Na^+^ concentration	137.90 (133.50, 141.50)	136.40 (133.08, 138.88)	-2.189	0.029*
CL^-^ concentration	101.60 (99.20, 103.90)	100.50 (97.63, 103.80)	-1.393	0.164
Liver function tests				
ALT (U/L)	21.00 (14.00, 33.50)	23.00 (15.00, 32.50)	0.594	0.552
AST (U/L)	28.00 (20.00, 40.00)	30.50 (19.75, 51.25)	1.339	0.181
Albumin (g/L)	35.70 (31.15, 39.4)	31.99 ± 0.80	-3.380	0.001*
Globulin (g/L)	34.80 (27.35, 41.35)	38.25 ± 3.05	-1.218	0.223
A/G	1.03 (0.82, 1.32)	0.99 ± 0.04	-1.202	0.229
Inflammatory markers				
CRP (mg/L)	26.26 (7.17, 64.94)	75.32 (41.59, 101.73)	5.123	<0.001*
ESR (mm/h)	58.00 (34.00, 90.00)	64.19 ± 4.84	0.451	0.652
Procalcitonin (ng/mL)	0.080 (0.025, 0.425)	0.185 (0.068, 0.785)	2.632	0.008*
Combined inflammatory markers				
Neutrophil-to-Lymphocyte Ratio	4.03 (2.33, 7.28)	6.14 (3.30, 14.60)	3.040	0.002*
Lymphocyte-to-monocyte ratio	2.21 (1.45, 3.31)	1.80 (1.15, 3.04)	-1.497	0.134
CRP/Albumin	0.74 (0.21, 2.06)	2.34 (1.03, 3.56)	5.200	<0.001*
Immunological detection				
CD4^+^ T-cell counts (cells/μL)	109 (46, 211)	37 (11, 104)	-4.496	<0.001*
CD8^+^ T-cell counts (cells/μL)	596 (295, 794)	258 (108, 548)	-4.746	<0.001*

The bold values and symbol* mean p value <0.05.

### Construction of a predictive model for treatment failure in patients with AIDS combined with TB via the LASSO Cox model

**Figure 2:**
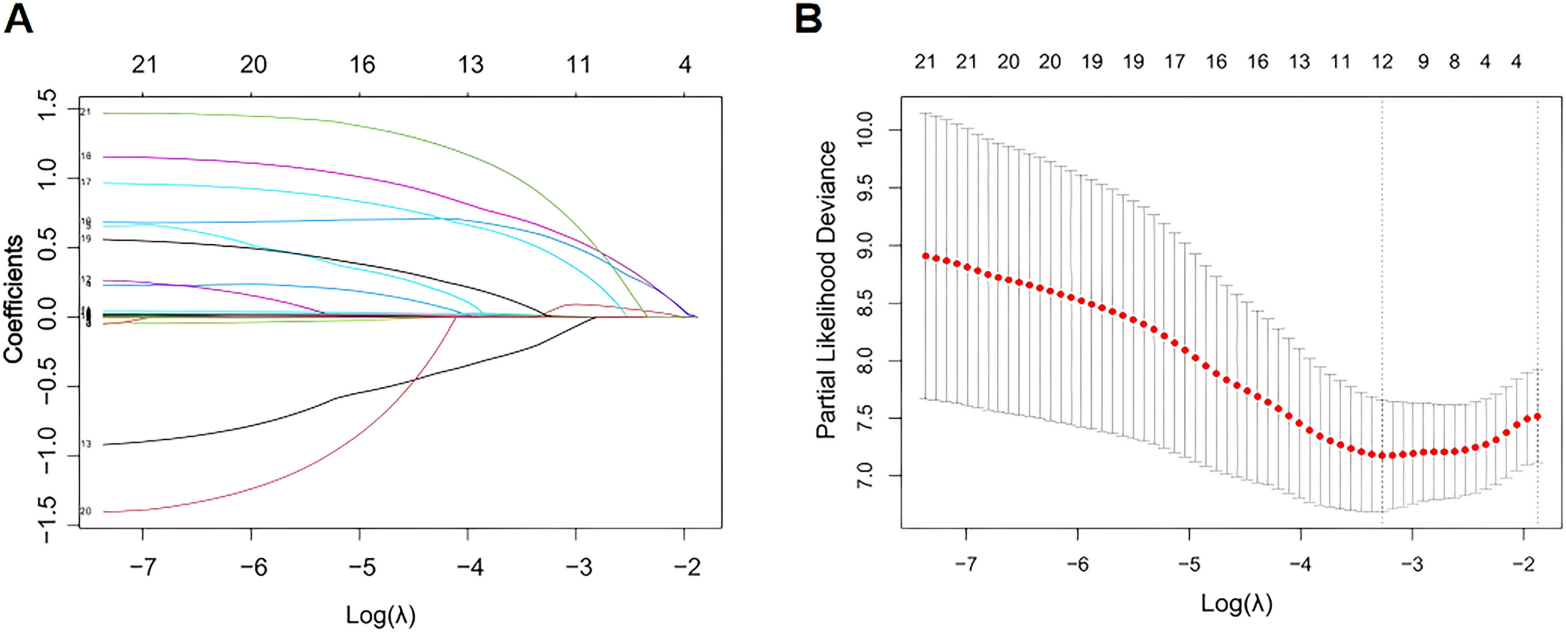
Variable selection via the LASSO Cox regression model. **(A)** Association between the log (λ) and regression coefficients of variables included in the LASSO analysis. **(B)** Process of screening the optimal λ value in the LASSO regression model by the 10-fold cross-validation method. LASSO, least absolute shrinkage and selection operator.

We randomly divided all enrolled participants into a training set and a validation set at a ratio of 2:1. The training set was used to construct the LASSO Cox model, and the validation set was used to test the accuracy and discrimination of the constructed model. Baseline variables with differences were included in LASSO regression analysis to identify potential prognostic factors affecting treatment outcomes in patients with AIDS combined with PTB. **Figure 2A** shows the variation characteristics of the regression coefficients of the variables included in the LASSO analysis. As the parameter λ increased, the regression coefficients were significantly compressed in the model, and the number of independent variables with zero coefficients gradually increased. The 10-fold cross-validation method was used to determine the optimal value of λ, and the results are shown in **Figure 2B**. In our study, the optimal model was chosen at λ= 0.038 (log λ= -3.27), and the variables screened by LASSO regression were subsequently included in the multivariate Cox analysis to establish the final model. **Table 2** shows the results of the multivariate Cox regression analysis. Our study revealed that CAR (hazard ratio (HR) 1.191, 95% CI 1.035–1.371, *p*=0.015), extrapulmonary disseminated tuberculosis (HR 2.008, 95% CI 1.044–3.866, *p*=0.037), other pulmonary infectious diseases (HR 2.447, 95% CI 1.235–4.850, *p*=0.010) and pulmonary cavitation (HR 2.760, 95% CI 1.147–6.643, *p*=0.024) were risk factors for the development of unfavorable outcomes in patients with AIDS combined with PTB. For every 1 increase in the CAR value in patients with AIDS combined with PTB, there was a 1.191-fold increase in the risk of treatment failure. Extrapulmonary disseminated tuberculosis increased the risk of treatment failure by 2.008 times. Patients with other pulmonary infectious diseases were 2.447 times more likely to have unfavorable outcomes. In addition, patients with pulmonary cavitation on chest imaging had a 2.76-fold increase in poor outcomes compared with patients without pulmonary cavitation. In contrast, CD4^+^ T-cell counts were a protective factor against the development of unsuccessful outcomes. Each 1 cell/µL increased in CD4^+^ T-cell counts was associated with a 0.4% reduction in the hazard of treatment failure (HR=0.996). The C-index was used to evaluate the predictive accuracy of the Cox model, and the results revealed that the C-index was 0.765 for the training set and 0.768 for the validation set.

**Table 2:** Multivariate Cox analysis of unfavorable outcomes in patients with AIDS combined with PTB.

Variables	Multivariate analysis		*P* value
	HR	95%CI	
CAR	1.191	(1.035-1.371)	**0.015** ^*^
Extrapulmonary disseminated tuberculosis			
Yes	2.008	(1.044-3.866)	**0.037** ^*^
No	1.000		
Other pulmonary infectious disease			
Yes	2.447	(1.235-4.850)	**0.010** ^*^
No	1.000		
Pulmonary cavitation			
Yes	2.760	(1.147-6.643)	**0.024** ^*^
No	1.000		
CD4^+^ T-cell counts(cells/μL)	0.996	(0.993-0.999)	**0.031** ^*^

The bold values and symbol* mean p value <0.05.

### Construction and validation of the nomogram

**Figure 3:**
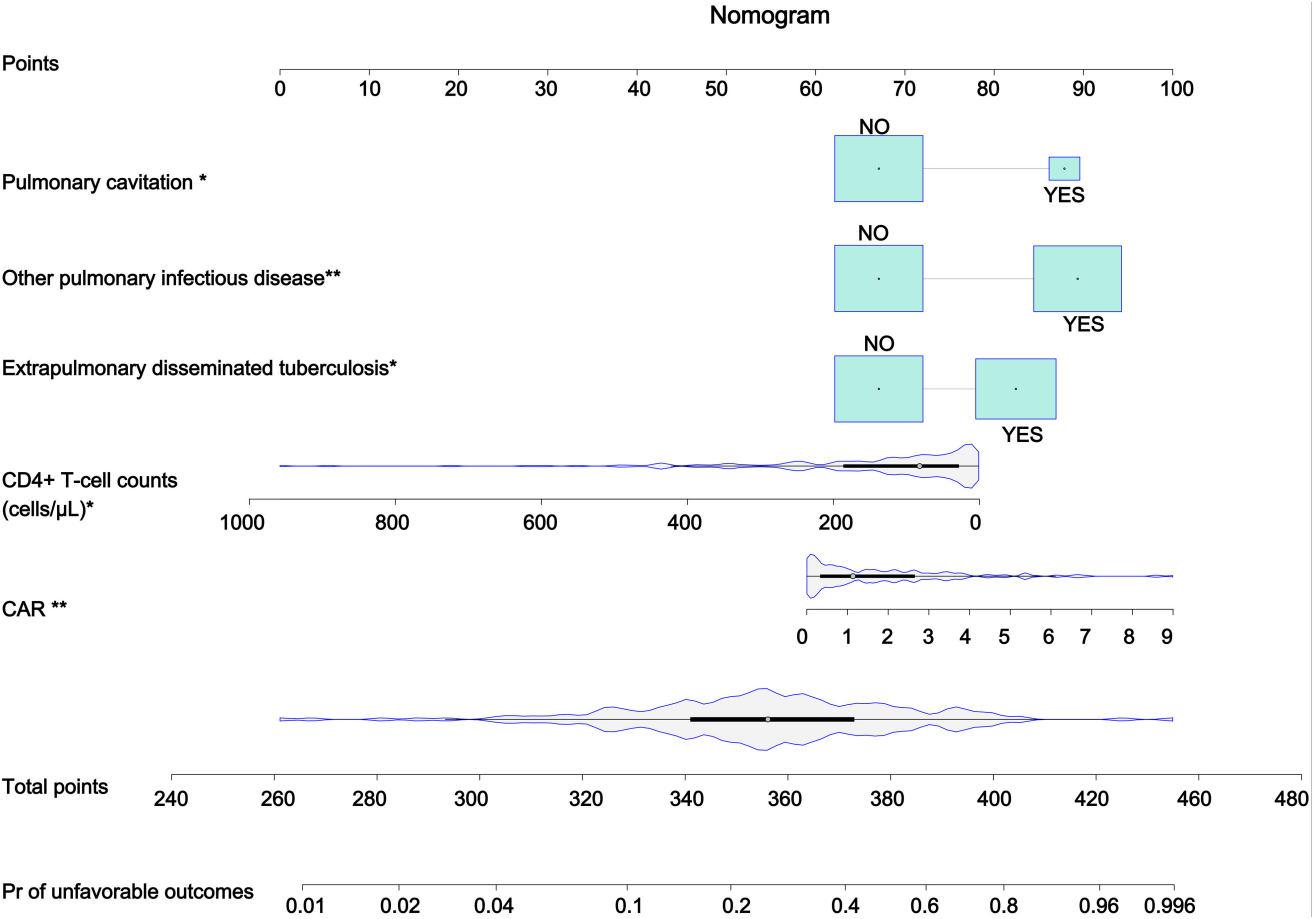
Nomogram for predicting unfavorable treatment outcomes in patients with AIDS combined with PTB. The predictive nomogram included the CAR, extrapulmonary disseminated tuberculosis, other lung infectious diseases, cavities in the lung and CD4^+^ T-cell counts. On the basis of a patient’s clinical characteristics and CAR and CD4^+^ T-cell counts values, we can calculate the total points and assess the patient’s risk of treatment failure by the probability of treatment failure corresponding to the total points. CAR, CRP/albumin ratio. The symbols * and ** represent variables that have a significant impact on unfavorable treatment outcomes, and the number of * reflects the contribution of the variables to the outcomes.

On the basis of the results of multivariate Cox regression analysis, we incorporated 5 variables, including the CAR, extrapulmonary disseminated tuberculosis, other pulmonary infectious diseases, pulmonary cavitation, and CD4^+^ T-cell counts, to establish a nomogram for predicting the risk of treatment failure in patients with AIDS combined with PTB (**Figure 3**). According to the magnitude of the regression coefficients of each indicator in the Cox model, a point was assigned to each indicator, and the points of each indicator were subsequently summed to obtain the total points. Considering the relationship between the total points and the probability of treatment failure, we could calculate the probability of treatment failure in patients with AIDS combined with PTB. In this study, the total risk score of the patients ranged from 240–480 points, and the probability of treatment failure within 12 months ranged from 0.01-0.996.

**Figure 4:**
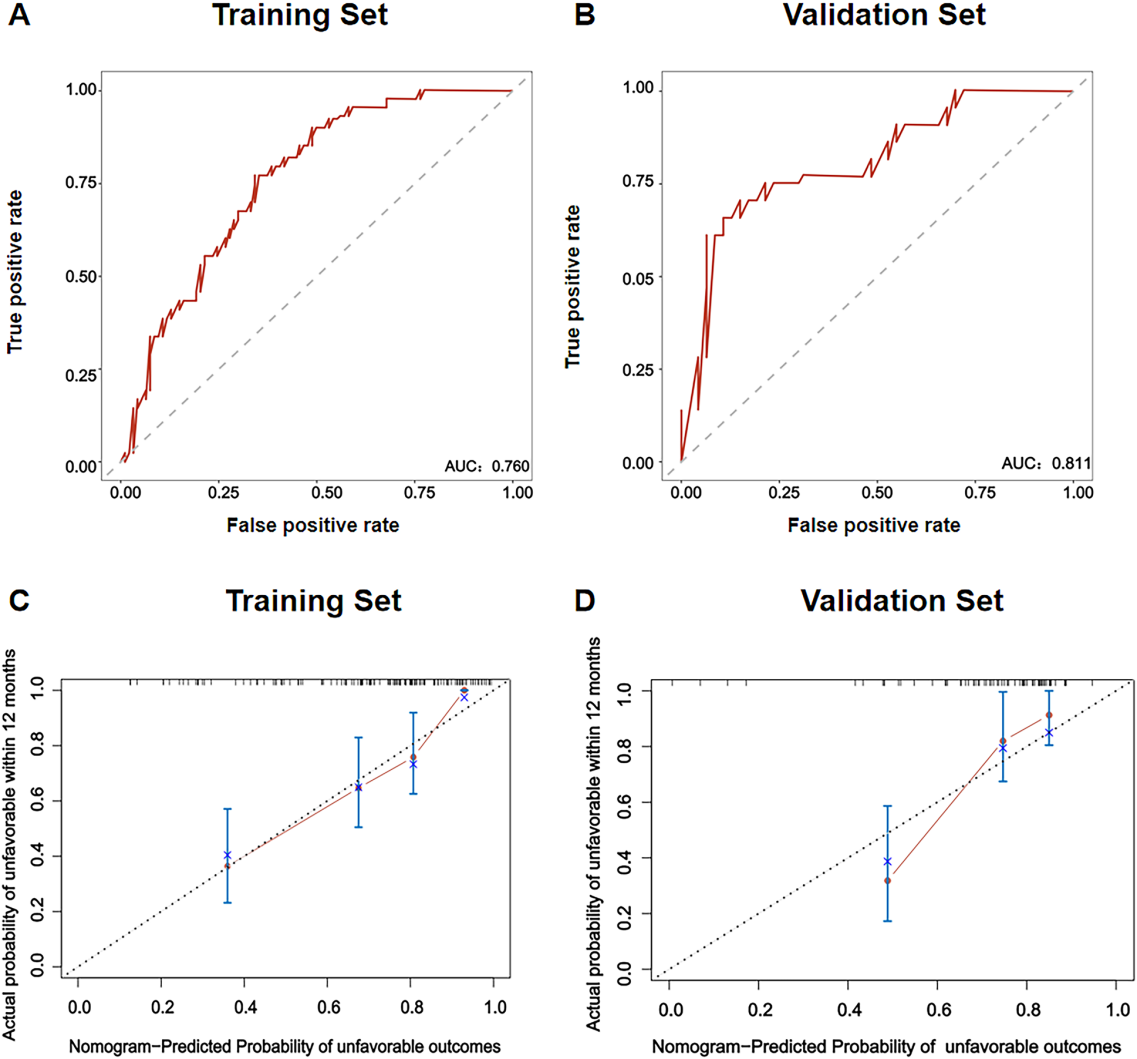
Validation of the nomogram. **(A)** ROC curve and AUC of the nomogram in the training set. **(B)** ROC curve and AUC of the nomogram in the validation set. **(C)** Calibration plot of the ability of the nomogram to predict unfavorable outcomes in patients with AIDS combined with PTB in the training set. **(D)** Calibration plot of the ability of the nomogram to predict unfavorable outcomes in patients with AIDS combined with PTB in the validation set. ROC, receiver operating characteristic curve; AUC, area under curve.

**Figure 5:**
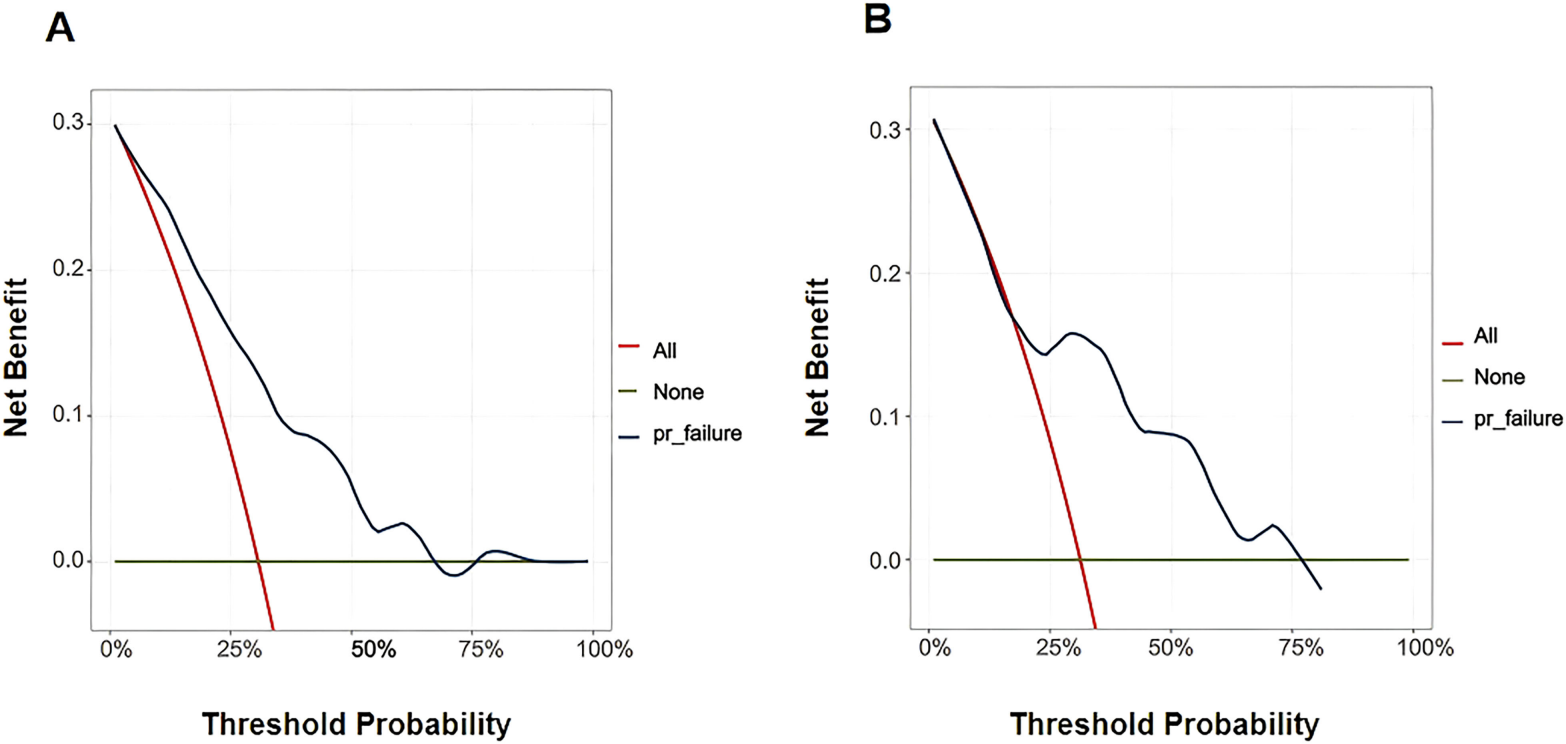
DCA plot of the nomogram. **(A)** DCA of the nomogram to predict unfavorable outcomes in patients with AIDS combined with PTB in the training set. **(B)** DCA of the nomogram to predict unfavorable outcomes in patients with AIDS combined with PTB in the validation set. DCA, decision curve analysis.

The performance of the nomogram was evaluated by the area under the curve (AUC), C-index, and calibration curve. As shown in **Figure 4A**, the AUC for predicting unfavorable outcomes in our study was 0.760. The discrimination ability of the model was assessed using the validation set, and the results revealed that the AUC was 0.811 in the validation set (**Figure 4B**). In addition, the C-index of the model was 0.765 for the training set and 0.768 for the validation set, which further confirmed the high discrimination ability of the nomogram. The calibration curves for the model for predicting the probability of treatment failure indicated high consistency between the predictions and the actual observations in both the training set and the validation set (**Figures 4C, D**). DCA was used to assess the clinical applicability of the nomogram. The DCA for the training set revealed that the net benefit of the model was high within a threshold probability range of 0.20 to 0.75 (**Figure 5A**). Similarly, the DCA for the validation set revealed that patients could benefit from the model when the threshold probability was in the range of 0.2 to 0.75 (**Figure 5B**).

### Risk stratification based on the nomogram

**Figure 6:**
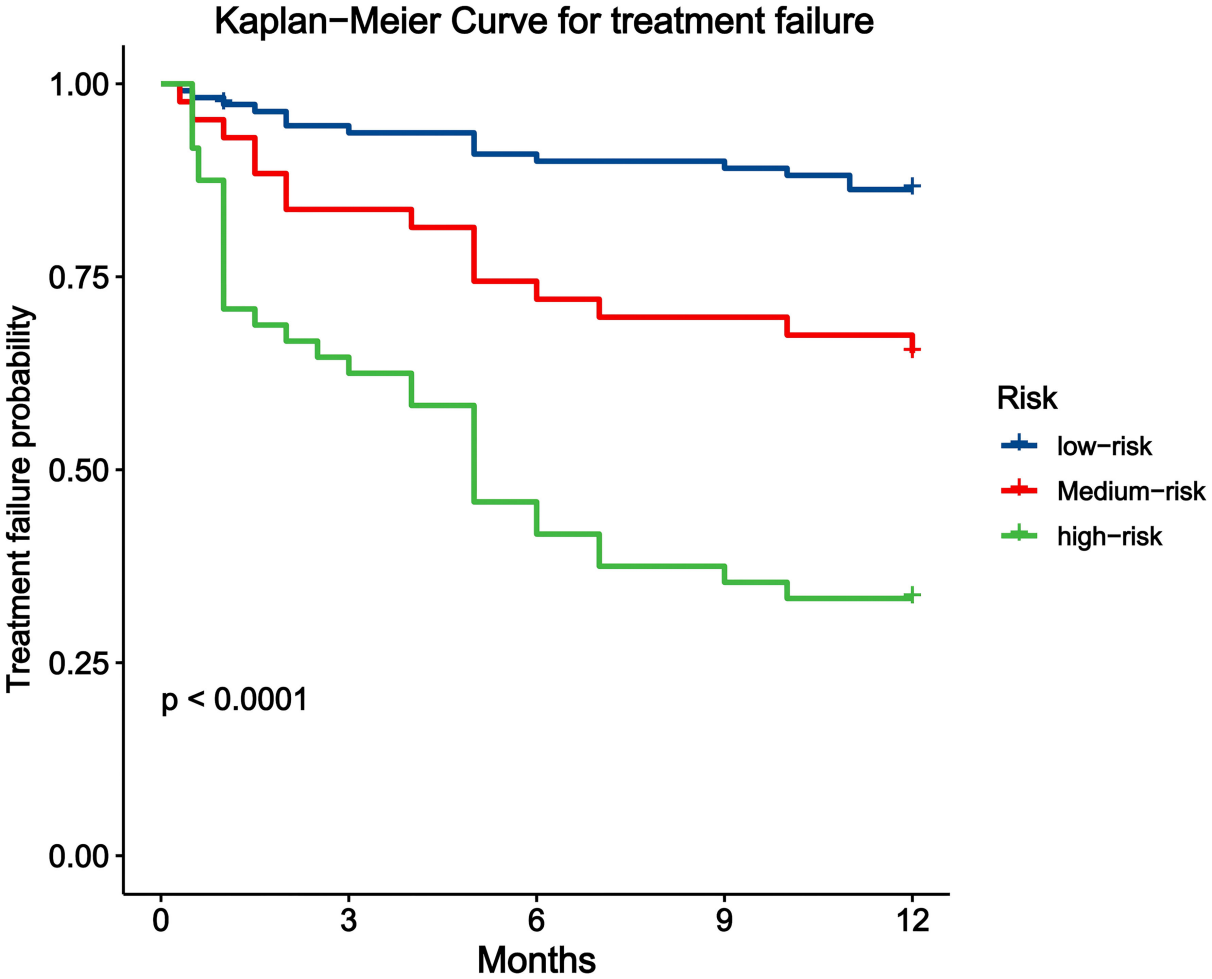
Kaplan-Meier curve of patients with different risk stratification.

To help clinicians better identify patients at high risk of treatment failure, we risk-stratified patients on the basis of the overall score of the nomogram according to the optimal cutoff value selected by X-tile software after completing the construction and validation of the nomogram. X-tile is a bio-informatics tool that can be used for biomarker evaluation and outcome-based cut-off value optimization. The software performs statistical tests using different values of the total score of the nomogram as cut-off values, and the result with the smallest *p* value is used as the optimal critical value (23). The results of risk stratification revealed that patients could be divided into three groups: the low-risk (total points <358), medium-risk (358 ≤ total points <373), and high-risk (total points ≥373) groups. K–M curves revealed large differences among the three groups of patients (**Figure 6**).

## Discussion

In this retrospective study, we developed a LASSO Cox regression model to identify several clinical predictors related to unfavorable treatment outcomes in patients with AIDS combined with PTB. Consequently, the CAR, extrapulmonary disseminated tuberculosis, other pulmonary infectious diseases, pulmonary cavitation, and CD4^+^ T-cell counts were identified and used to establish a prognostic nomogram. A validation study of this prognostic nomogram using the C-index, AUC and calibration curves demonstrated that it had good discrimination and calibration abilities. Additionally, we risk-stratified the patients according to the total points of the nomogram score, and subsequently, the patients were categorized into three groups: the low-risk, medium-risk, and high-risk groups. K–M curves revealed significant differences among the three groups of patients. This nomogram can help clinicians identify patients with AIDS combined with PTB who are at risk of treatment failure at an early stage, and individualized management and regular drug resistance testing of patients at medium or high risk play important roles in reducing the incidence of relapse and drug-resistant TB.

AIDS combined with TB places a heavy burden on global health care systems, and timely diagnosis and effective treatment are key to achieving global tuberculosis epidemic control. However, the management of patients with AIDS combined with TB is extremely complex due to several factors, including delayed diagnosis and treatment, interactions between ART drugs and anti-TB drugs, drug resistance, and concerns about adherence to treatment (28, 29). Data from the *Global Tuberculosis Report 2024* showed that globally, the treatment success rate for TB patients treated with first-line anti-TB drugs is 88%, but that for HIV-infected patients is only 79% (4). In this study, the treatment success rate of anti-TB therapy in patients with AIDS combined with PTB was 69.5%, which was lower than that reported by the WHO. This may be because the individuals included in this study were all inpatients and had relatively low CD4^+^ T-cell counts and a high incidence of extrapulmonary tuberculosis and opportunistic infections such as PCP. In addition, the anti-TB treatment success rate reported in this study was similar to that reported in Ethiopia (69.9%) (30) and higher than that reported in Malaysia (53.4%) (31) and Cameroon (60.8%) (32). This discrepancy may be due to differences in socioeconomic status, the quality of health services, and the prevalence of health education across countries. In summary, HIV coinfection has a significant effect on TB treatment outcomes; therefore, countries should pay more attention to AIDS combined with TB to achieve the WHO goal of ending the TB epidemic by 2030.

LASSO is a special penalized least squares method. This method introduces a tuning parameter λ to compress the regression coefficients of certain variables to zero for the purpose of variable selection and model adjustment. LASSO has good performance in dealing with multicollinearity data and in improving model prediction accuracy (33). Suresh S et al. showed that LASSO had higher predictive accuracy in improving the predictive performance of different training fractions using the high-dimensional breast cancer datasets containing survival endpoints compared to other regularized regression methods, such as ridge regression or elastic net (34). The Cox proportional hazard regression model is a classic approach to survival analysis and has several advantages over other models. First of all, the HR and coefficients of the Cox model output variables can directly quantify the effects of covariates on outcomes, thus facilitating clinical decision making. Second, Cox model can be directly transformed into a nomogram for visualizing individualized risk scores, which is more convenient for clinical application. However, “black box models” such as the Random Forest model and the Neural Network model are not the best choices in visualizing the contribution of variables (9). A Previous study showed that the prediction accuracy of the LASSO Cox model was higher than that of the LASSO Support Vector Machine model and the LASSO Random Forest model, confirming the higher application value of the LASSO Cox model in clinical outcome prediction (34). Therefore, we developed a LASSO Cox model to identify prognostic factors for unfavorable anti-TB treatment outcomes and constructed a predictive nomogram to assess the risk of unfavorable outcomes in patients with AIDS combined with PTB.

Our study revealed that extrapulmonary tuberculosis is an independent risk factor for treatment failure in patients with AIDS combined with TB. Similarly, a study from the Netherlands reported that combined extrapulmonary tuberculosis was a significant predictor of unsuccessful treatment outcomes in TB patients (35). This could be partly explained by the increased risk of death in patients with extrapulmonary tuberculosis. Studies have shown that combined extrapulmonary tuberculosis is an independent risk factor for mortality in HIV/*Mtb* co-infected individuals (36, 37). As a result of immunosuppression, patients with AIDS combined with PTB often have extrapulmonary tuberculosis, including central nervous system tuberculosis and lymphatic system tuberculosis, which further exacerbates damage to the immune system of patients with AIDS and leads to disease progression and related deaths. In addition, a longer course of anti-TB treatment in patients with combined extrapulmonary tuberculosis may lead to an increased incidence of adverse effects and drug resistance, with a corresponding increased risk of treatment failure. Our study revealed that comorbidity with other pulmonary infectious diseases is a risk factor for treatment failure in patients with AIDS combined with PTB. When a patient is immunocompromised, *Klebsiella pneumoniae, Staphylococcus aureus*, and opportunistic infectious agents such as *Pneumocystis carinii* invade the respiratory system and interact with *Mtb*, further exacerbating lung inflammation and even leading to respiratory failure and death. In addition, invasion of the respiratory system by *Klebsiella pneumoniae* and *Staphylococcus aureus* destroys the structure of the lung tissue, leading to insufficient local concentrations of anti-TB drugs, which in turn affects the efficacy of anti-TB treatment. Therefore, when initiating anti-TB treatment, clinicians should assess patients for comorbid extrapulmonary tuberculosis and other pulmonary infectious diseases to reduce the risk of treatment failure.

Pulmonary cavitation in patients with TB is caused by necrosis and expansion of tuberculous granulation or diseased tissue, which are closely associated with transmission and poor treatment outcomes (38). Studies have shown that the risk of treatment failure and relapse is significantly increased if cavities are observed radiographically during the first two months of treatment (39). This may be explained by insufficient local concentrations of anti-TB drugs due to the poor penetration of anti-TB drugs into avascular cavities (40). Pulmonary cavitation can also lead to the development of drug resistance, thus affecting the effectiveness of anti-TB treatment. Owing to the high levels of oxygen in the cavity, *Mtb* proliferates rapidly, which increases the frequency of replication-induced mutations and the development of drug resistance (41–43). Additionally, cavities can lead to permanent destruction of lung tissue and may become a habitat for other pathogens, which may worsen patient conditions. Consistently, we found that pulmonary cavitation is an independent risk factor for treatment failure in patients with AIDS combined with TB, which may further strengthen previous conclusions.

The CAR is a biomarker reflecting the inflammatory status of an organism and has the clinical advantage of being easily accessible, as CRP and albumin levels are widely used in medical health care centers. Previous studies have confirmed that the CAR can be used as a predictive marker for cardiovascular disease, sepsis, and COVID-19 (44–47). Similarly, our study revealed that higher CAR may be associated with the risk of treatment failure in patients with AIDS combined with TB, which can be partly explained by the abnormal inflammatory status of these patients. Previous studies have shown that a decrease in CRP levels is positively correlated with the degree of lung involvement and sputum culture conversion rates; thus, CRP levels may be a potential marker for predicting the outcome of anti-TB treatment (48). Albumin, one of the two main components of serum proteins, is an important marker associated with inflammation and infection. Various studies have shown that the serum albumin concentration is closely related to the prognosis of patients with tumors and chronic infectious TB (49, 50). Simona Stefanescu et al. reported that the CAR was significantly lower in patients whose sputum culture results were negative after 2 months of anti-TB treatment and that the CAR may be a better predictor than the CRP or albumin level alone (51).

Our study revealed that CD4^+^ T-cell counts were an independent protective factor against unsuccessful treatment outcomes in patients with AIDS combined with PTB. Higher CD4^+^ T-cell counts are associated with a lower risk of anti-TB treatment failure in patients with AIDS combined with PTB. Consistent with the present results, a study from India revealed that patients with CD4^+^ T-cell counts less than 50 cells per cumm had a 3–4-fold increased risk of poor prognosis and that CD4^+^ T-cell counts were strongly associated with all-cause mortality in HIV/*Mtb* co-infected individuals (52). CD4^+^ T-cell counts play an important role in the anti-TB immune response of the host by secreting IFN-γ, which promotes macrophage activation and generates an inflammatory response, thereby enhancing macrophage clearance of *Mtb* (2). When CD4^+^ T-cell counts decrease, the host anti-*Mtb* immune response decreases, which increases the multiplication of *Mtb* and prevents granulomas from limiting *Mtb.* Consequently, the progression and dissemination of *Mtb* ultimately increase the risk of drug resistance and death.

The nomogram constructed in this study can help clinicians quickly identify patients with AIDS combined with PTB who are at risk of treatment failure, which plays an important role in assisting in clinical decision-making and achieving precision treatment. When a patient with AIDS combined with PTB presents to the clinician, the clinician should evaluate the patient for the presence of extrapulmonary tuberculosis, pulmonary cavitation, and other pulmonary infectious diseases, as well as having a test for the patient’s CD4^+^ T-cell counts and CAR. Once the relevant assessments have been completed, the patient’s risk points can be obtained by bringing the relevant data into our model. Patients with a total point of more than 358 are considered to be at risk for unfavorable treatment outcomes, and should be provided with more comprehensive education on medication adherence and the importance of anti-TB treatment. Clinicians should also intensify clinical follow-up to assess drug resistance and efficacy of treatment and to reduce the risk of anti-TB treatment. Our model has several advantages. First, the five indicators used in this model are easily accessible in the clinic. Second, our model showed good discrimination ability and consistency on the basis of the AUC, C-index, and calibration curves.

This study also has several limitations. First, the sample size of our study was relatively small and focused on a single medical organization; thus, the generalizability of this model to other health care institutions and other regions may weak. Therefore, future validation in a large multicenter cohort is needed. Second, as the individuals enrolled in this study were all inpatients, the included patients had a relatively severe disease state, which may have led to an overestimation of the incidence of unfavorable treatment outcomes. In addition, an overrepresentation of hospitalized patients may introduce selection bias. We will include more outpatients to verify the accuracy and validity of the model constructed in this study in the future. Third, we did not evaluate socioeconomic factors or other factors that may result in loss in follow-up, which are critical for the assessment of treatment outcomes and the design of effective intervention strategies. Finally, this study was retrospective and cannot be used to prove causality. In addition, retrospective studies may have some inherent bias, including the effects of partially unnoticed, unmeasured bias and confounding factors (53). Despite these limitations, we explored the risk factors affecting treatment outcomes in patients with AIDS combined with TB and constructed a predictive model with a good discrimination ability and consistency. We hope that this model will help clinicians rapidly identify patients at risk of treatment failure, thereby effectively reducing the incidence of relapse and drug-resistant TB.

## Conclusion

In conclusion, our study identified five variables related to unfavorable anti-TB treatment outcomes through the LASSO Cox model. The CAR, extrapulmonary disseminated tuberculosis, other pulmonary infectious diseases, and pulmonary cavitation resulted in an increased risk of treatment failure in patients with AIDS combined with PTB, whereas an increase in the CD4^+^ T-cell counts reduced the risk of treatment failure. Notably, we constructed a nomogram to assess the risk of unsuccessful treatment outcomes in patients with AIDS combined with PTB and categorized patients into high-risk, medium-risk, and low-risk groups on the basis of the nomogram. This can help clinicians quickly identify patients at greater risk of treatment failure, thereby assisting in clinical decision-making.

## Data Availability

The original contributions presented in the study are included in the article/supplementary material. Further inquiries can be directed to the corresponding author.
